# Collaboration during a crisis – the Lighthouse Lab volunteers

**DOI:** 10.1099/mic.0.000960

**Published:** 2020-07-28

**Authors:** Fatima R. Ulhuq, Sophia K. Berry, Lucy Kelly, Ben Stansfield, Anna Deal, Harriet Lester

**Affiliations:** ^1^​ Microbes in Health and Disease Theme, Newcastle University Biosciences Institute, Newcastle University, Newcastle upon Tyne, NE2 4HH, UK; ^2^​ Department of Veterinary Medicine, University of Cambridge, Cambridge, CB3 0ES, UK; ^3^​ School of Life Sciences, Gibbet Hill Campus, University of Warwick, Coventry, CV4 7AL, UK; ^4^​ Research Institute for Sport and Exercise Sciences, Liverpool John Moores University, Liverpool, L3 3AF, UK; ^5^​ Department of Physiology, University of Arizona College of Medicine, Tucson, Arizona, 85721, USA; ^6^​ Institute for Infection and Immunity, St George’s, University of London, London, SW17 0RE, UK; ^7^​ Department of Biochemistry, University of Oxford, Oxford, OX1 3QU, UK

**Keywords:** Lighthouse Lab, COVID-19, volunteers, collaboration, teamwork, pandemic

## Abstract

As a group of early-career researchers, we recount our experiences of volunteering at one of the national Lighthouse Labs based at the UK Biocentre in Milton Keynes. We worked together as part of a multidisciplinary team to support the large-scale processing of coronavirus disease 2019 (COVID-19) swabs from across the whole of the UK.

On 11 March 2020, the World Health Organization (WHO) declared the coronavirus disease 2019 (COVID-19) outbreak as a pandemic and the UK was then put into lockdown on 23 March 2020. Two weeks into lockdown, scientists from across the country received a message from the Microbiology Society on behalf of the UK Government. It was a call for scientists to volunteer in one of the three national Lighthouse Labs based in Milton Keynes, Cheshire and Glasgow testing COVID-19 samples. Like many in the scientific community, we felt a sense of helplessness during the lockdown, and an urge to do our bit to help with the national pandemic response, so we applied for the role without hesitation. Following a telephone interview, within 48 h we were invited to attend an induction day at the UK Biocentre Lighthouse Lab in Milton Keynes. The following evening, we started on our very first 12 h night shift together.

Prior to lockdown, we were all based at different universities and were at different stages in our scientific careers: Dr Fatima Ulhuq (postdoctoral research associate, Newcastle University); Dr Sophia Berry (postdoctoral research associate, University of Cambridge); Lucy Kelly (PhD student, University of Warwick); Harriet Lester (DPhil student, University of Oxford); Anna Deal (Research Assistant, St George’s, University of London) and Ben Stansfield (MSc student, Liverpool John Moore’s University). As university labs had closed their doors to non-essential work during the pandemic, we were unable to continue our lab-based research projects. Like so many others in our position, we used this lab downtime as an opportunity to work on other areas of our research, attend online training courses and prepare manuscripts for publication. In addition, many of us also signed up to community volunteer groups and the NHS volunteer responders programme. However, when the opportunity arose, we felt strongly that our time and the lab skills gained during our master’s and PhD studies would be best utilized working in the Lighthouse Labs. Making this move was a big decision as we were unsure of how long we would be expected to work, what exactly the job would involve and how it would affect our current ‘day jobs’. However, we each received great support and encouragement from our supervisors and universities, who arranged secondment contracts for staff and allowed flexible working for others.


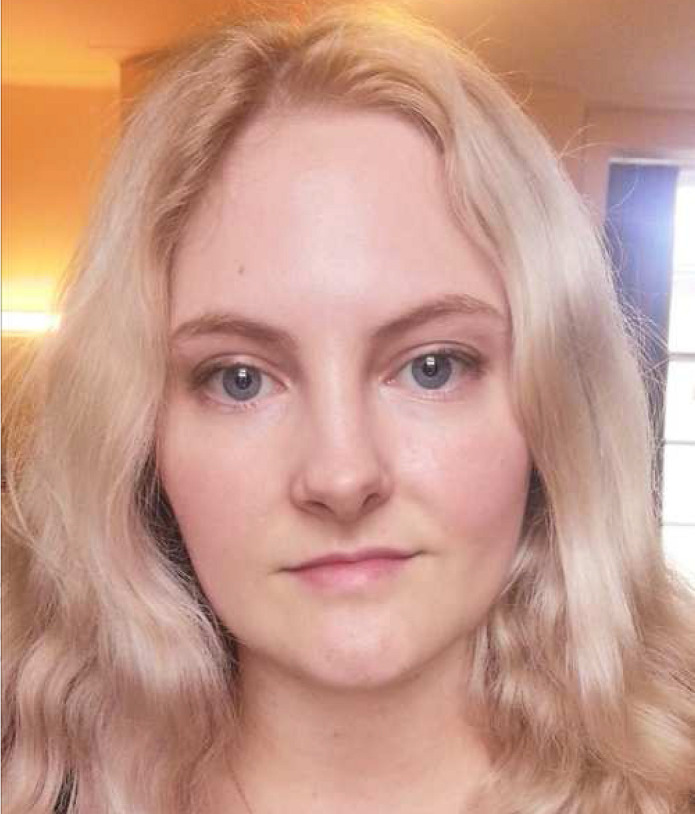


‘I got an email from the biochemistry department that was asking for volunteers at the Lighthouse Lab in Milton Keynes. I was quite frustrated staying at home because I wanted to be out actively helping people. My mother died of COVID-19 shortly after I signed up, which really cemented the fact that I needed to actively fight the pandemic.’ – Harriet Lester

Many of the volunteers at the Lighthouse Lab arrived from all over the UK and were offered temporary accommodation at the Holiday Inn, which allowed volunteers to stay overnight between shifts. However, for volunteers living further afield, commuting was not an option and so we were provided with accommodation for the full duration of our contract, which in some cases meant moving away from home for a number of months. Moving to Milton Keynes indefinitely in the middle of a global pandemic was daunting for many, but we were well supported during our time working in the lab, from accommodation and travel expenses, to being provided food on shift, and this made our transition to Milton Keynes seamless.

When we first started working at the Lighthouse Lab we were amazed by the sheer scale of the facility and the army of volunteers working there. The Lighthouse Lab is a 24 h testing operation, and operates in two 12 h shifts of 40 volunteers each plus support staff. The COVID-19 testing process is split into the following workstations: unpacking the nose and throat swabs, sample preparation, RNA isolation, PCR preparation and qPCR. Most of the scientists specialized in one workstation, which allowed us to be trained to perform tasks in our section with a high degree of accuracy and efficiency.

Nose and throat swabs from home testing kits, drive-through testing sites and care homes were delivered to the Lighthouse Lab throughout the day and night by Royal Mail. Once unpackaged, the first part of the testing procedure was to inactivate the virus using a lysis buffer. This involved working in a biosafety cabinet in order to safely open the test kit containing the swab. The sample barcode was recorded and the viral transport medium pipetted into an allocated well of a 96-well plate containing lysis buffer. Every step of the manual process had to be witnessed and verified by at least two pairs of eyes. As the volume and frequency of Royal Mail deliveries increased, in order to increase testing capacity we began shifting to automated sample preparation using Tecan liquid handling robots. This involved manually loading swab tubes into racks, programming a Tecan to record the barcode of each swab and transfer viral transport medium from each tube to wells of the 96-well plate. A Tecan robot could transfer eight viral samples at once, greatly increasing the speed at which a plate could be processed. Whilst volunteers working in pairs could manually process around six plates per 12 h shift, a Tecan robot operated by two volunteers could process 30 plates. It is undoubtedly this automation that enabled our lab to process tens of thousands of tests a day by the end of April.

Once the virus was inactivated, the samples were mixed with magnetic beads and the viral RNA was extracted using a KingFisher automated extraction instrument. The eluted RNA was then transferred to the next workstation: PCR preparation. Tecan robots were used to add each sample of extracted RNA to the qPCR mastermix. Finally, RT-qPCR was performed to determine if any severe acute respiratory syndrome coronavirus 2 (SARS-CoV-2) viral RNA was present. The number of tests we received each day varied, but with this system in place we were able to process up to 30,000 samples within a 24 h period. This could not have been achieved without UK universities pulling together and providing essential equipment for the running of these Lighthouse Labs.


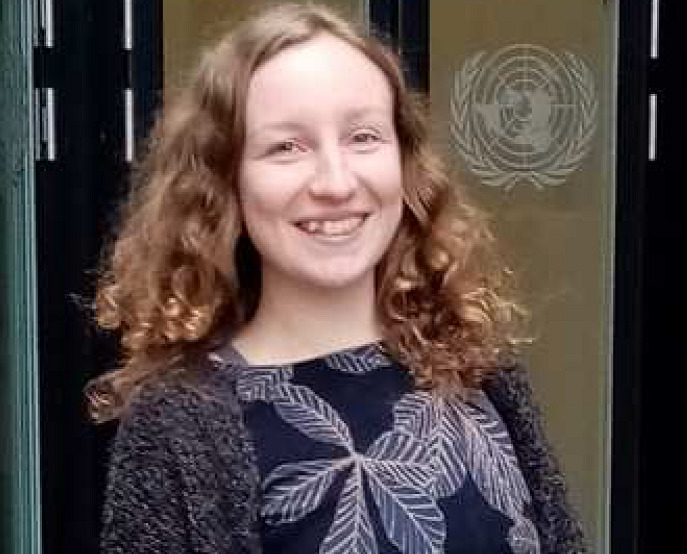


‘There was a very strong sense of community and common purpose that drove us all forward during the long shifts.’ – Anna Deal

Working at the testing facility was a big change from our day jobs as PhD students, postdocs or research assistants, where we are accustomed to having complete autonomy over our projects. Working at the Lighthouse Lab was a much more collaborative effort, with an emphasis on teamwork. The other major difference was the importance of the job at hand. Behind every swab we processed, there was a patient at home displaying symptoms and anxiously awaiting their result. Knowing that every sample belonged to a patient whose life could be reliant on us doing our job correctly was an added pressure we had not experienced before, and one that will forever change our outlook on future work.


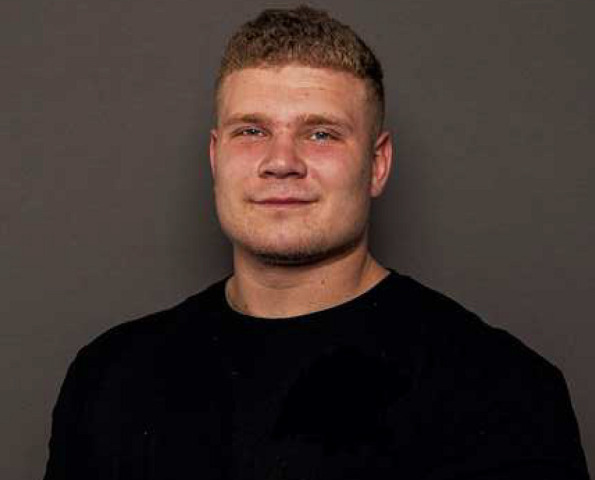


‘No matter the background, experience or qualifications everyone was on the same level and the camaraderie between everyone was brilliant. I have made friends for life whilst working at the Lighthouse Lab.’ – Ben Stansfield


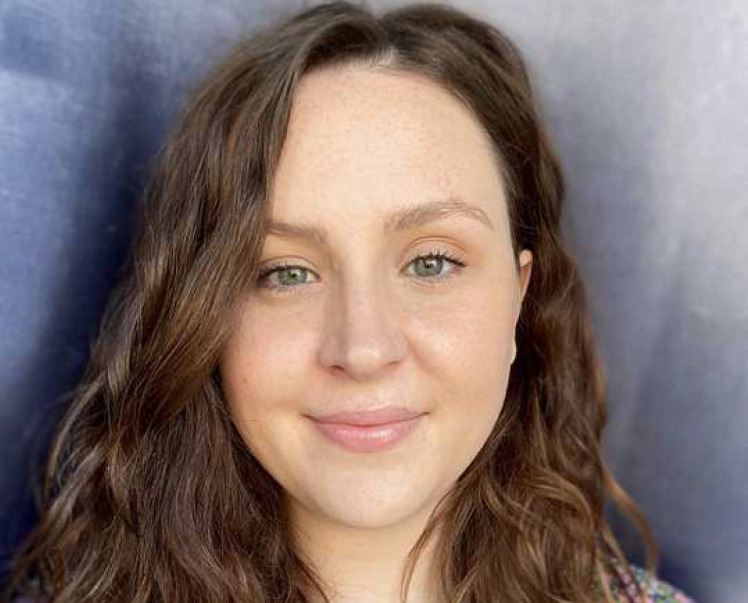


‘The experience showed me the importance of surrounding yourselves with great colleagues, if you have a supportive team then you can get through the tough work without any issues.’ – Lucy Kelly

In unprecedented times, each volunteer at the Lighthouse Lab made a conscious decision to offer their skills and experience to make a difference, and this united us. From day one, we were thrown into a room full of people with different backgrounds, and yet from the beginning everyone was on the same page. Day to day, we could be working with undergraduates, postdocs, industry scientists, vets, professors, group leaders, lab managers and more, which meant that every day was a chance to get a different perspective on science, as well as life in general. We felt lucky to have had the chance to meet and work with so many different scientists from varied backgrounds during our time at the diagnostic lab, which is a unique opportunity in academia as you often only work closely with those in your field.

The collaborative nature of the work meant we got to know each other very quickly and really pulled together as a team. We worked 12 h shifts both day and night under a huge amount of pressure, but the people working alongside us were the shining lights of the lab. We worked hard, and any issues we encountered were discussed and resolved as a team. We helped each other out whenever we could, and we made the work enjoyable.


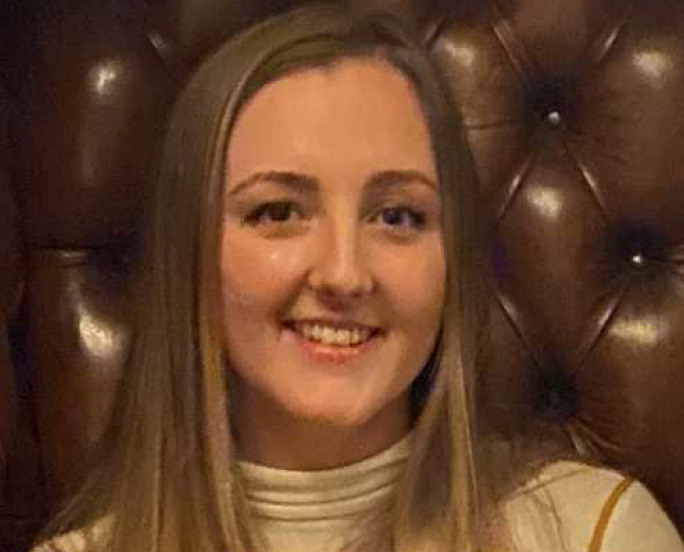


‘I am proud to have worked alongside so many great scientists as a part of the national effort tackling COVID-19. I have undoubtedly left the Lighthouse Lab with a network of connections and friendships that will be long lasting’. – Dr Sophia Berry


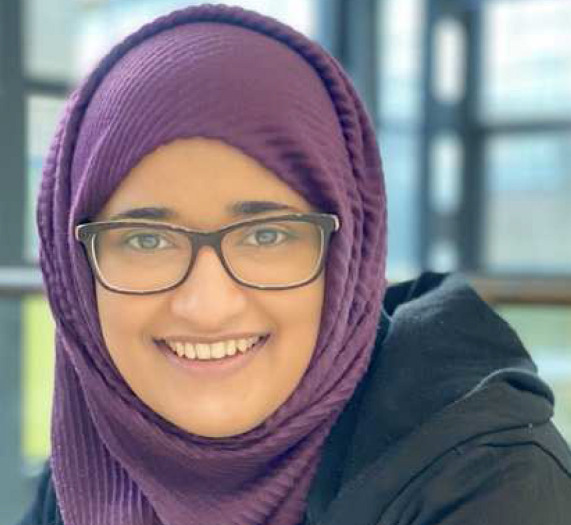


‘This exemplified for me how scientific research should be carried out - collaboratively within a diverse workforce. This is what truly drives innovation and helps to produce stronger research.’ – Dr Fatima Ulhuq

Academia can be somewhat solitary at times, something that is deemed the ‘norm’. However, moving forward this has opened our eyes to how vital collaborative work is, not only for the advancement of research but also for life experiences. Great science can be achieved when many great minds are involved. It has been a tremendous honour and a privilege to play a small part in helping our country during this difficult time.

